# Predicting the Health Condition of mHealth App Users with Large Differences in the Number of Recorded Observations - Where to Learn from?

**DOI:** 10.1007/978-3-030-61527-7_43

**Published:** 2020-09-19

**Authors:** Vishnu Unnikrishnan, Yash Shah, Miro Schleicher, Mirela Strandzheva, Plamen Dimitrov, Doroteya Velikova, Ruediger Pryss, Johannes Schobel, Winfried Schlee, Myra Spiliopoulou

**Affiliations:** 8 University of Bari Aldo Moro, Bari, Italy; 9 Aristotle University of Thessaloniki, Thessaloniki, Greece; 10 Open University of Cyprus, Nicosia, Cyprus; 11 Dalhousie University, Halifax, NS Canada; 12 Otto-von-Guericke University Magdeburg, Magdeburg, Germany; 13 National Center for Public Health and Analyses, Sofia, Bulgaria; 14 University of Würzburg, Würzburg, Germany; 15 Ulm University, Ulm, Germany; 16 University of Regensburg, Regensburg, Germany

## Abstract

Some mHealth apps record user activity continuously and unobtrusively, while other apps rely by nature on user engagement and self-discipline: users are asked to enter data that cannot be assessed otherwise, e.g., on how they feel and what non-measurable symptoms they have. Over time, this leads to substantial differences in the length of the time series of recordings for the different users. In this study, we propose two algorithms for wellbeing-prediction from such time series, and we compare their performance on the users of a pilot study on diabetic patients - with time series length varying between 8 and 87 recordings.

Our first approach learns a model from the few users, on which many recordings are available, and applies this model to predict the 2nd, 3rd, and so forth recording of users newly joining the mHealth platform. Our second approach rather exploits the similarity among the first few recordings of newly arriving users. Our results for the first approach indicate that the target variable for users who use the app for long are not predictive for users who use the app only for a short time. Our results for the second approach indicate that few initial recordings suffice to inform the predictive model and improve performance considerably.

## Introduction

Recent trends in consumer electronics towards affordable and relatively powerful devices capable of sensing health-related attributes have been matched by an increase in research interest in exploiting this data to assist the healthcare practitioner. Not only do these devices help in diagnostics, by recording values of attributes related to health and subjective well-being; they also allow that the disease may be monitored with only asynchronous involvement of the practitioner. Self-monitoring of the disease contributes thus to patient empowerment, and also delivers precious data that can be used for personalization, i.e. for treatments tailored to the individual needs and characteristics. This potential requires adequate data to build upon.

A major challenge of mobile health platforms that collect user inputs is that the amount of data users contribute can vary substantially. As we reported in [15] when analysing user recordings on the mobile health platform “TrackYourTinnitus” [9], a minority of users interact intensively with the system and contribute a disproportionately large amount of data, while the majority of users contribute very few inputs. In this study, we investigate whether predictions can be made for this majority of users by learning a model on the few users who provide many data to the system. Also, differently from our work in [15], we focus here on a one-step-ahead forecast instead of classification.

We propose an approach that learns from users who contribute long sequences of inputs to predict the subjective perception of wellbeing for users who contribute only short sequences of input data, including users that have very recently joined the platform. Each user in the system is required to fill in a “End of Day Questionnaire”, where he reports among other things the overall “feeling in control”, the variable of prediction interest. These user-level timestamped observations therefore constitute one user-centric time series, the length of which varies depending on how long the user has been in the system, and how the doctor’s recommendation of filling in the questionnaire at the end of every day has been followed. We denote the set of users with long sequences of recordings as $U_{long}$ and the users with few recordings as $U_{short}$. Our approach deals with the following three questions:

- RQ1: How well can we predict the behaviour of users in $U_{short}$ given the data from the users in $U_{long}$?
- RQ2: Can we predict the entire sequence of observations of a user in $U_{short}$ with a model trained only on data from users in $U_{long}$? (i.e, does a model learned on data from users with long sequences transfer to those with short ones?)
- RQ3: How can we incorporate early recordings of users in $U_{short}$ incrementally into the model to improve predictive performance?

The paper is organised as follows: Sect. 2 introduces related literature, followed by Sect. 3, which introduces the m-Health application on which the this work is based. Section 4 discusses our proposed solution, followed by a discussion in Sect. 5, and closing remarks in Sect. 6.

## Related Work

In our work, we concentrate on time series in applications of health and wellbeing. The early study [5] by Madan et al. reported on the potential of mobile technologies to capture epidemiological behaviour change, including physiological symptoms like running nose, and mental health conditions like stress. For example, they found that total communication of the affected persons decreased for the response “sad-lonely-depressed” (cf. [5] for the definition of this response). While a change in communication intensity can be captured by Bluetooth connection activity or absence thereof, the information on how a person feels demands user inputs. Ecological Momentary Assessments (EMA) are a widespread tool for this purpose [3, 14].

EMA is an instrument for assessing “behavioral and cognitive processes in their natural settings” [14]. From the technical perspective, EMA recording is feasible and well-supported. For example, in their survey on sleep self-management apps [2], Choi et al. list the recording of user-entered data as an important functionality, and stress that all investigated apps do support this functionality. However, next to the technical modalities, EMA relies also on self-discipline and adherence.

As Mohr et al. stress in [6], “although a number of small studies have demonstrated the technical feasibility of sensing mood, these findings do not appear to generalize”. In the meanwhile, there are large studies involving EMA recordings of more participants for longer time periods. However, the emphasis seems still to be on users who interact intensively with the mobile health application. In their insightful comparison of the results of EMA recordings with the TrackYourTinnitus mHealth app versus retrospective ratings of the users, only users with at least 10 days of interaction were considered [11]. For findings with the TrackYourStress platform that records EMA geolocation, only users with at least 10 recordings per day were considered [10].

This provokes the question of whether users with few recordings belong to the same population as users with many recordings. In [8], Probst et al. considered both users with few days of recordings and users with many days of recordings for their Multi-Level Analysis (median number of days: 11, with range from 1 to 415 days), but demanded at least 3 EMA per day, each of them containing answers for the three EMA items under study [8]. In this work, we do not attempt to win insights that pertain to a specific group of users, but rather to assess whether the EMA of users with few recordings can be predicted by models learned on users with many recordings.

The EMA of mobile health app users constitute multivariate time series. The challenge posed by short time series is discussed by Palivonaite and Ragulskis in their work on short-term forecasting [7], where they associate the length of the time series to the reliability of longer-term forecasts.

Dynamic Time Warping (DTW) or one of its numerous enhancements can be used to compare time series of different lengths and exploit their similarity for learning. DTW is a very old method, cf. [16], for an early citation to DTW by authors Yfantis and Elison who proposed a faster alternative. Such methods can be used to enhance algorithms like [1, 15], which do predictions by building a model for each time series, but can also exploit information from similar time series. Despite this potential, the amount of data per user in some mHealth applications is very small, so that we opt for similarity-based methods that capitalize more on the similarity of values than on the ordering of the values – albeit both are taken into account.

## EMA with the TrackYourDiabetes Mobile Health App

**Fig. 1.**
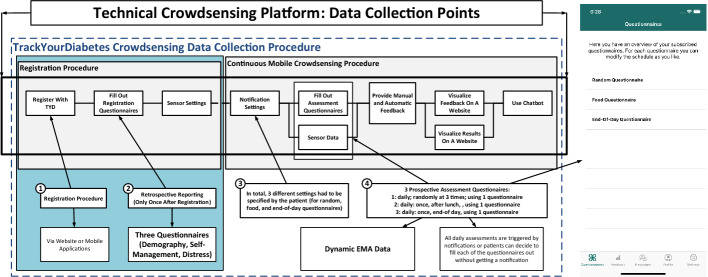
Mobile crowdsensing collection procedure of TrackYourDiabetes

As part of two pilot studies on empowerment of diabetes patients, a mobile crowdsensing framework was adjusted to implement the TrackYourDiabetes mHealth platform [4, 9]. Figure 1 summarizes the entire procedure of the app from the patient’s point of view. The pilot studies were conducted in regions of Spain and Bulgaria, and involved patient recruitment and exposition to two variants of the app, while under remote supervision by a practitioner.

The platform comprises two mobile applications (i.e., native Android and iOS apps), a relational database, a RESTful API [12], and a web application. The mobile applications are only used by the patients, while the web application was used by the patients as well as their related healthcare professionals. The latter were enabled by the web application to monitor the data of the patients as well as to provide individual feedback if wanted or required.

Before starting interaction with the app, study participants registered with the platform by using the mobile apps or a web application  . After that, they have to fill out three registration questionnaires once  : one registration questionnaire collects demographic data, one collects information on the self-management of the patients with his/her diabetes, and one captures the extend to which diabetes causes distress to the patient.

There were EMA recordings more than once a day, concerning physical activity and food intake, and EMA recordings at the end of each day, using the End-of-Day questionnaire items depicted in Table 1. Furthermore, individualised messages based on given answers of daily assessments were provided with the goal to better motivate the patients in using the platform. The healthcare professional(s) responsible for the participants could also provide individualised feedback. Finally, a chatbot was integrated, which could be used by the patients to discuss questions on their diabetes. For the analysis of the proposed approach, we concentrated on the Bulgarian pilot study and investigated solely the user inputs to the End-of-Day questionnaire; no further features were considered.

**Table 1.** Variables in the dataset: questions in the end-of-day questionnaire

#	Question	Set of answers/Data type
01	How often do you have measured your sugar level today?	Numeric
02	For how many minutes have you performed physical activity or sports today?	Numeric
03	How many bread units have you eaten today?	Numeric
04	Did you have signs of hyper- or hypoglycemia today?	“Don’t know”, “No”, “Both”, “Hypoglycemia”, or “Hyperglycemia”
05	Did you feel to be in control of your diabetes today?	Numeric, [0–100]

## Our Method

We investigate a prediction problem on timestamped data, transferring a predictor learned on the data of one set of users, $U_{long}$, to another set of users $U_{short}$. In all cases, our goal is to predict many observations of a user, not just the next one, as is typical in many time series prediction problems.

### Core Concepts and Core Elements

This section offers a brief overview of the terms used in this work and their exact definitions, which is followed by a broader description of our workflow in Sect. 4.2.

*User Sequences:* Each user *p* who uses the mHealth app generates a time-ordered sequence of observations $x_{p,t}$, where *p* is the user, and *t* denotes time.

We distinguish between users with *short sequences* of observations, constituting a set $U_{short}$, and users with *long sequences* of observations, constituting a set $U_{long}$. For the partitioning of users into these two strata, we consider a threshold $\tau _{length}$.

In our experiments, we set $\tau _{length}$ on the basis of the user-observations distribution, which has shown a gap. In distributions that follow a power law, $\tau _{length}$ serves to separate between the short head and the long tail. More generally, we may decide to place into $U_{short}$ those users who have very recently started their interaction with the app and thus have contributed only few initial observations.

*Observations:* An observation is a multi-dimensional vector of values from a feature space *F*. In our application scenario, an observation is an EMA recording comprised of answers to questions from a questionnaire. Accordingly, an observation is a mix of numerical and categorical variables.

*Handling Categorical Data: A term Frequency-Inverse Document Frequency (TF-IDF) Inspired Approach:* Before training the models, it is important to consider the exact way in which categorical attributes in the input data are used. Of the various questions in the questionnaire answered by the users, the questions that generate categorical data (chosen from a drop-down list) need to be treated to accommodate for the fact that not all answers are equally likely. Compared to simply using a standard method like one-hot encoding, this step brings the answer closer to the user’s history, for e.g., by more accurately capturing the information that a user who commonly answers a question with “no” has said “yes”, even if “yes” frequently appears in the dataset.

We treat the answers to this categorical data as ‘words’, and each session where the questionnaire is answered as a ‘document’. During preprocessing, given the exact answer chosen by a user during a particular day, we replace the binary flag marking the presence of that word with a new value that is adjusted to reflect the amount of “surprise” in seeing that data point given the user through the use of the TF-IDF (see [13]) inspired formula: $preprocessed\_value = f_{term} \cdot -log\frac{n_{term}}{N}$, where $f_{term}$ can only be binary, since the categorical answer only picks one term from a list of several. The inverse document frequency component measures how often the term has appeared in the user history.

*Core Learning Method:* Given data $P_p$ for all users $p \in U_{long}$ for time points $1 \ldots t$, we have $P_p = \{x_{p,1} \ldots x_{p,t}\}$. Using this data, we create a linear regression model that for each possible $i \in {1 \ldots t-1}$, learns to predict the target variable for time point $i+1$. Naturally, since there is no known label for the last time point *t*, each user *p* with a sequence length of *t* only provides $t\,-\,1$ time points of training data. This model is only used for predicting the labels for the observations $\{x_{p,1} \ldots x_{p,t-1}\}$ for all users $p \in U_{short}$.

*Augmented Method:* We augment the above method by creating predictions specific to the users $U_{short}$: In addition to the above model which only learns on the users of $U_{long}$, we add an additional K-NN Regression model that is trained only on the user’s own history of observations. This means that given an observation $x_{p,t}$, we can generate predictions for $x_{p,t+1}$ from two models, the model trained on all users in $U_{long}$, and additionally the K-NN Regression model that has only been trained on the observations of the user *p* seen so far, i.e. $x_{1} \ldots x_{t-2}$ (Note: The training data for $p \in U_{short}$ ends at $t-2$, because $x_{t-1}$ is used as the label for the training point $x_{t-2}$, and the true label for $x_{t-1}$ has not been observed yet).

### Prediction Workflows

**Proof-of-Concept Step:** The basic workflow we propose has a preliminary step and two components. The preliminary step is designed to check that the task is indeed *learnable*, and success at this stage can ensure that the further steps in the workflow are applicable. For this, instead of training a model on only data from users in $U_{long}$, a model is trained on 75% of all data, and the performance is analysed to confirm that the model can learn given the data. By framing the problem as a regressor and not as a time series forecast, we avoid the problem of having insufficient data to train a time series forecasting model. This model can unfortunately not be used as a baseline to compare against since it does not learn on the same amount of data as the model learning only on $U_{long}$, and also because the number of data points available for testing over users in $U_{short}$ is very small (often only a couple of observations). However, the performance of this model can still be considered a benchmark for the upper limit of performance for the transfer learning model.

**Fig. 2.**
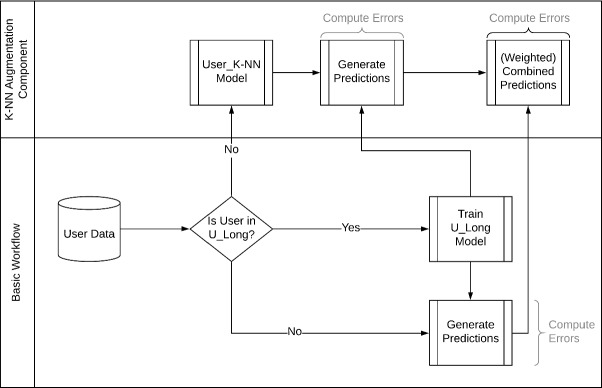
Prediction workflows

**Basic Workflow:** In this workflow, we find a subset of the dataset *D* comprising of only the data $x_{p,t}$, where $p \in U_{long}$. This creates a model trained only on the data from users with long sequences, the performance of which is tested on users of $U_{short}$. It is important to remember that the model has the challenging task of making predictions for users that have never been seen by the system, and predictions for them are made based only on what has been learned over the user. This is arguably more challenging than predicting unseen observations for users who have already contributed observations to the training set. Additionally, since these users have not adhered to instructions of the physician to use the application for the prescribed period of 2 months, it is possible that these users differ somehow in the expression or the perception of the disease in some way. However, it is still possible that a model learned on those data points from long users bring a modest predictability to the disease development of users in $U_{short}$. Similarly to the model introduced above, we learn to predict the numeric value of the target variable for the next observation given the questionnaire answers of the current observation (including the current value of the target variable). A graphical overview of the workflow is shown under ‘Basic Workflow’ of Fig. 2.

**K-Nearest Neighbour (K-NN) Augmented Workflow:** If the users in $U_{short}$ are indeed different from the users in $U_{long}$, then using a model that transfers the parameters learned on $U_{long}$ is not expected to bring reliable predictions to the users in $U_{short}$. However, since the users in $U_{short}$ do not bring enough data to train complex models, only simple techniques can be used to try and incrementally improve predictive performance over users in $U_{short}$ by capturing the idiosyncratic patterns in the user’s disease development/answering style. This design aims to balance the tradeoff between keeping as much data as we can use to learn about how the disease develops, while also staying close to the idiosyncratic ways in which the user may answer questions. In this work, we propose the use of a K-Nearest Neighbours regressor trained over the user’s own history, the predictions of which are used to augment the predictions from the $U_{long}$ model weighted on their past errors (similarly to [1]). Restricting the K-NN regressor to the user’s own sequence also has the unintended consequence of out-of-the-box support for data-privacy, something that is especially relevant in the medical domain.

During use, the K-NN regressor is incrementally trained on the user sequence as more of it becomes available, and the errors are recorded for comparison to the standard $U_{long}$ model. Figure 2 shows an overview of the model training process with the K-NN augmentation component.

## Results

We describe the dataset of our evaluation in Subsect. 5.1, and then explain in Subsect. 5.2 how the number of users with short and long sequences affect the prediction tasks and the settings of K-NN in the augmented workflow. We evaluate using Mean Absolute Error (MAE). The results of the *proof-of-concept* experiment are in Subsect. 5.3, while the results for the basic workflow and the KNN-augmented workflows are in Subsect. 5.4.

### The Dataset

For our evaluation, we used the dataset of the Bulgarian pilot study. This dataset contains observations from 11 study participants. While the inclusion of the users from the pilot study in Spain is desirable, a model that learns on the combined data of the two pilots is not done for two reasons: (a) The two countries are different in the dominant diabetes type that the users have, and (b) Many users in the Spain pilot use continuous blood sugar measuring devices, strongly influencing the accuracy of the “self-assessed” blood sugar estimations, and therefore, the “feeling in control”. We set $\tau _{length}=30$ days, whereupon 6 of the users belong to $U_{long}$ (30+ days) and 5 users are in $U_{short}$ (8–13 days) after eliminating users with 5 users with less than 3 days of data. We denote this dataset as L6+S5_dataset hereafter, to stress the number of users per length-stratum.

**Fig. 3.**
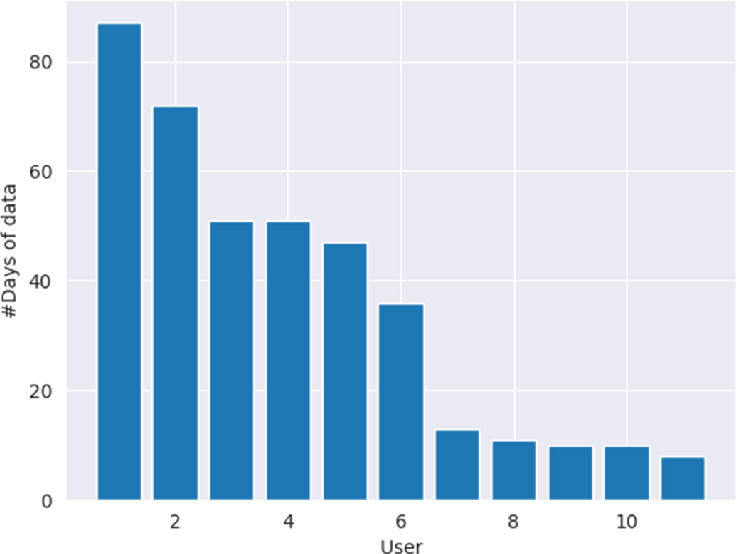
Number of days with EOD observations per user; user #1 is the user with the largest number of EOD observations, user #11 has the smallest number

Figure 3 depicts the number of days of interaction for all users. It can be observed that there is a clear separation between users in $U_{long}$ compared to the rest of the users.

Of the 5 variables of the EOD questionnaire filled by the pilot study participants (cf. Sect. 3), the target variable is the 5th one on Table 1, i.e. each user’s self-reported ‘feeling in control’, on a scale of 0 to 100. We denote this variable as ‘EOD_feel’ hereafter.

### Prediction Tasks and Imposed Restrictions on Training

For the proof-of-concept step in Subsect. 4.2, we train a predictor on the first 75% of the observations of the users in $U\_{long}$ of L6+S5_dataset and predict the subsequent 25% observations. As can be seen on Fig. 3, the 6 users in $U_{long}$ contribute unequally to learning: user #1 contributes more than 60 (out of ca. 85) observations to the training dataset, while user #6 contributes less than 30 (ie half as many). Similarly, we predict the EOD_feel value of more than 20 observations of user #1 and ca. 10 of user #6.

For the basic workflow of Subsect. 4.2, the prediction task is to predict *all* observations of the 5 users in $U_{short}$ of the L6+S5_dataset, without having seen any observations on them during training. This amounts to 47 predictions.

For the K-NN augmented workflow, some observations of each user in $U_{short}$ of the L6+S5_dataset are disclosed and used for augmentation of the model learned on all of the $U_{long}$ observations in the L6+S5_dataset. User #7 has less than 15 observations, user #11 has 8 (cf. Fig. 3). This imposes an upper limit to *K*: if we set $K=8$, we cannot do any predictions on user #11. On the other hand, K-NN based regression needs at least 2 observations per user to learn.

Larger values of *K* allow for a more robust regression model and make the prediction task easier, since less EOD_feel values are predicted. To investigate whether the very few first observations on a user can inform a model learned on $U_{long}$, we have set $K=2$. This amounts to 37 predictions.

### Learning a Predictor on $U_{long}$: Proof-of-Concept Experiment

**Fig. 4.**
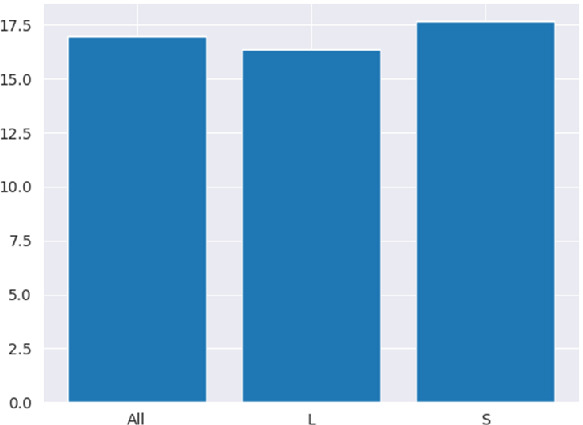
MAE in $U_{long}\,\cup \,{}U_{short}$ (All), in $U_{long}$ (S) and in $U_{short}$ (S)

The Goal of this experiment is to check whether the prediction problem is indeed *learnable*, in the sense that we can derive a useful prediction model. Figure 4 shows the performance of the proof-of-concept regression model for the first prediction task of Subsect. 5.2 on L6+S5_dataset, learning on the first 75% of all user observations (All), and accordingly on the first 75% of the observations in $U_{long}$ (L), resp. $U_{short}$ (S).

For “All” (leftmost part), MAE remains around 17%, decreasing slightly within “L” ($U_{long}$) and increasing slightly within $U_{short}$. However, MAE within “S” ( $U_{short}$) is rather unreliable, since there are less than two observations per user in the testset (more precisely: 1.4). Hence, these MAE values serve only as lower limit for the errors of the transferred models.

### Learning on Users in $U_{long}$ to Make Predictions for Users in $U_{short}$: Transfer Learning Experiments

Since we have a baseline (albeit weak, since errors for $U_{short}$ are not reliable) for the performance of a model on the data from all users, we can now investigate the transfer learning case where the model is only learned on the users of $U_{long}$. As already described above, there are two workflows that use such a model, a more basic workflow that uses a model learned over $U_{long}$ only, and another model that augments the basic workflow with a user-specific K-Nearest observations regressor. The models are all evaluated against the absolute errors they make in their predictions. The ‘mean’ in the Mean Absolute Error may either be computed over all predictions that a model has made, or may be restricted to the predictions for particular users. Given the rather short sequence lengths of the users in $U_{short}$, it is necessary to not rely on point-estimates like means, but consider the ‘spread’ in the errors as well. We therefore present box plots over all the prediction errors for the basic and K-NN Augmented workflow. The entire test set contains 47 observations for which predictions are required.

**Fig. 5.**
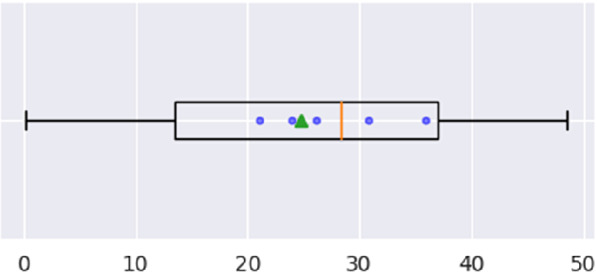
Basic workflow: box plot of errors for predicted next-observation EOD_feel, along with mean (Green Triangle), median (Yellow Line), and user-level MAEs (blue dots) for all users in $U_{short}$. (Color figure online)

**Basic Workflow.** In this workflow, instead of learning a model over data from all users, as described in Sect. 5.3, we will learn a model only on those users who have contributed more than 30 days of data, the necessary criterion for their addition to the set $U_{long}$. Figure 5 shows a box plot of the absolute prediction errors for the transferred model. Compared to the basic model in Sect. 4.2, the MAE over all predictions for all users has increased from 17.5 to 24.76 (indicated by the green triangle). However, since not all users in $U_{short}$ have the same sequence lengths, the MAE is biased towards the users with longer sequences. The blue dots inside the box plot shows the MAE for each user separately, and we can see that the users who are best predicted have errors as low as 21, with the worst-predicted users showing MAE in excess of 35. The mean being closer to 21 indicates that the well-predicted users are indeed the ones with longer sequences. This indicates that they are more similar to the users in $U_{long}$ than other shorter members of $U_{short}$.

**Fig. 6.**
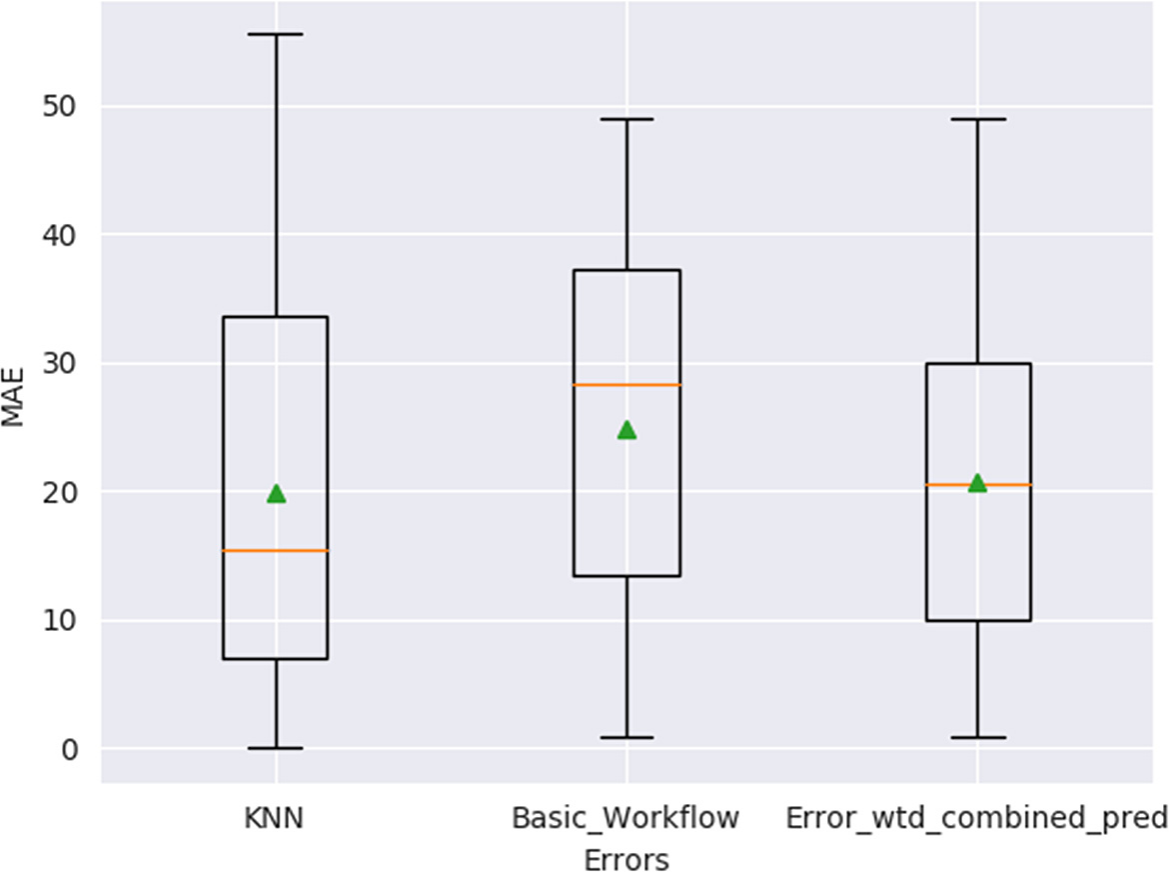
K-NN Augmented workflow: bloxplots of absolute errors for KNN model, basic workflow, and the combined workflow. Means are denoted by the green triangle and medians by the yellow horizontal line (Color figure online)

**K-NN Augmented Workflow:** This section discusses the results for the more advanced “K-NN Augmented Workflow” detailed in Sect. 4, where a user-level model learned on data from $U_{short}$ augments the predictions of the model discussed above. Figure 6 shows a box plot of the absolute prediction errors for the K-NN Regressor, along with comparisons against the $U_{long}$ model’s errors. The box plot on the far right shows the errors in the case where the predictions of each method are combined as a weighted average on their cumulative errors for the user to form a final prediction. Since the users in $U_{short}$ can have as few as 8 observations, our choice of *K* is quite strongly limited to very low numbers, as the K-NN Regressor cannot create predictions until it sees at least *K* observations. In these cases, the K-NN is assumed to make the same prediction as the Linear Regression model over $U_{long}$, since it is necessary to compare the errors of the two models for the same number of predictions.

It can be seen in Fig. 6, the K-NN model does indeed show lower mean and median errors, indicated by the green triangle and the line in the box plot. However, it can also be seen that the worst-case performance of the K-NN model is worse than that of the Linear Regression model. The roughly similarly sized gaps between the mean and the median errors in the K-NN and the Linear Regression models indicate that both models sometimes make large errors, albeit in different directions. Combining the predictions from both models does seem to mitigate these worst-case errors, since the mean and the median absolute errors are observed to be very close, at around 20.

**Fig. 7.**
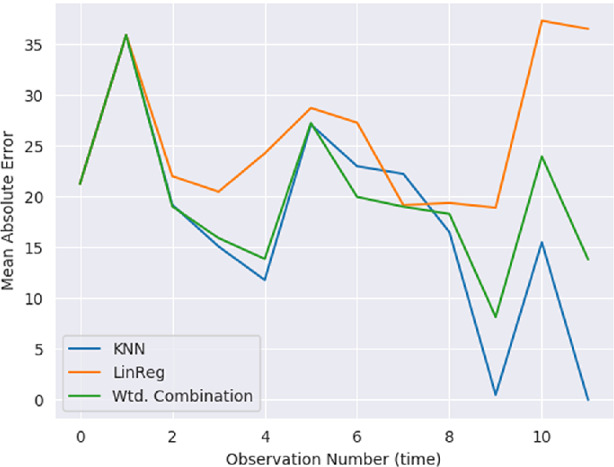
K-NN augmented workflow: development in error with time

In addition to the boxplots of the error itself, Fig. 7 shows how the error develops over time for users in $U_{short}$ as they stay longer in the system. The X-Axis shows the observation number, with the MAE on the Y-axis. The MAE at each time point is averaged over the individual prediction errors over all users at that time point. Until the $K^{th}$ observation, the K-NN predictor does not generate predictions, but we have used the linear regression model prediction errors in order to not unfairly favour any algorithm. From the $3^{rd}$ observation, however, we see that the user-level K-NN predictor almost always outperforms the linear regression model (and therefore the weighted average model). It is also noteworthy that until the 7th time point, the error-weighted combination of both models is very close to the K-NN model. This shows that augmenting the predictions of the basic workflow with the K-NN regressor does improve performance. The results beyond the 8th observation get progressively less reliable since all users in $U_{long}$ have at least 8 observations, but the number of users contributing to the averages after time point 8 get unreliable, though it is possible that users in $U_{short}$ are more and more predictable given the history of their own observations with time.

## Conclusions and Closing Remarks

In this work, we studied if the data from users of a diabetes self-management app with more than 30 days of data ($U_{long}$) can be used to infer something about the future of less intensive users with less data. Since neither the number of patients ($N=11$) nor the number of observations for the longest-sequence user (87) is very long, we investigate simpler models like linear regression. The model is trained to predict the next observation for user-reported “feeling in control”, the last question of the *End Of Day* questionnaire, given the answers to all questions of End Of Day questionnaire for the current observation. The categorical information in the dataset is handled using a method inspired by TF-IDF to capture the ‘surprise element’ in an answer given a user. i.e., when a user answers a question like (s)he usually does, that answer gets a smaller weight than if the answer is unexpected.

Further, we investigate whether transfer learning can be used to learn a model on the users of $U_{long}$ in order to make predictions for the observations of users in $U_{short}$. We saw that the transferred model predictably shows a higher error, which can be mitigated by combining the predictions of the $U_{long}$ model with a K-Nearest Neighbours Regressor over the patient’s own past data. The short sequences necessitate that the *K* is limited to quite low values, but the predictor that combines the predictions of both models does eliminate some extreme errors, bringing the mean and the median errors closer.

The primary threat to validity of this work is the size of the dataset from which the conclusions have been drawn. The large disparity between the lengths in $U_{long}$ and $U_{short}$ make further analysis of the K-NN Augmented predictor less reliable, making the findings more qualitative than quantitative. Although two pilots exist from which data can be analysed, this study focused the investigation only on data from Bulgaria because the users for the two studies are drawn from different populations (the proportion of Type 2 diabetics is very different, and the Spanish pilot users had continuous glucose monitoring devices implanted). Additionally, the mHealth application collects more data from the users, of which the EOD questionnaire is only one. A system with either more users or longer observation sequences may enable the study of how other dimensions not measured by the EOD questionnaire may affect the subjective “Feeling in control”, or allow for the use of more sophisticated models than simple linear regression. It is also highly likely that $x_{t}$ might not be best predicted by the value of $x_{t-1}$, but rather by some larger or even user-dependent lag, depending on external factors like weekends, or user-specific factors like exercise routine. The testing of this parameter is challenging at the moment because it further decreases the amount of data available for testing the predictions over users in $U_{short}$, or adds more features and complexity in the context of already scarce data. If such a large disparity did not exist between the lengths of users in $U_{long}$ and $U_{short}$, it would also be possible to investigate the aspects that characterise users who transition from $U_{short}$ to $U_{long}$.
